# Evolution of SARS-CoV-2-specific CD4^+^ T cell epitopes

**DOI:** 10.1007/s00251-023-01295-8

**Published:** 2023-01-31

**Authors:** Marina Brand, Can Keşmir

**Affiliations:** Theoretical Biology & Bioinformatics, Utrecht University, Utrecht, Netherlands

**Keywords:** CD4 T cell epitopes, Bioinformatics, Vaccination, VOCs, Conservation, SARS-CoV-2

## Abstract

Vaccination clearly decreases coronavirus disease 2019 (COVID-19) mortality; however, they also impose selection pressure on the virus, which promotes the evolution of immune escape variants. For example, despite the high vaccination level in especially Western countries, the Omicron variant caused millions of breakthrough infections, suggesting that the highly mutated spike protein in the Omicron variant can escape antibody immunity much more efficiently than the other variants of concern (VOCs). In this study, we investigated the resistance/susceptibility of T helper cell responses that are necessary for generating efficient long-lasting antibody immunity, in several VOCs. By predicting T helper cell epitopes on the spike protein for most common HLA-DRB1 alleles worldwide, we found that although most of high frequency HLA-DRB1 alleles have several potential T helper cell epitopes, few alleles like HLA-DRB1 13:01 and 11:01 are not predicted to have any significant T helper cell responses after vaccination. Using these predictions, a population based on realistic human leukocyte antigen-II (HLA-II) frequencies were simulated to visualize the T helper cell immunity on the population level. While a small fraction of this population had alarmingly little predicted CD4 T cell epitopes, the majority had several epitopes that should be enough to generate efficient B cell responses. Moreover, we show that VOC spike mutations hardly affect T helper epitopes and mainly occur in other residues of the spike protein. These results suggest that lack of long-lasting antibody responses is not likely due to loss of T helper cell epitopes in new VOCs.

**Supplementary Information:**

The online version contains supplementary material available at 10.1007/s00251-023-01295-8.

## Introduction

The severe acute respiratory syndrome coronavirus 2 (SARS-CoV-2) pandemic has caused over 6 million deaths worldwide (World Health Organization 2022). Coronavirus disease 2019 (COVID-19) severity ranges from mild symptoms to severe illness in a small fraction of patients. Smoking, advanced age, sex, and comorbidities such as hypertension, diabetes, or obesity are associated with increased disease morbidity and mortality (Callender et al. 2020). Moreover, the presence of human coronavirus (HCoV)–induced cross-reactive T cells may contribute to clinical protection (Lipsitch et al. 2020; Braun et al. 2020), although its longevity remains questionable (Saletti et al. 2020). A central question during the pandemics has been the underlying mechanisms causing the variation of disease severity in SARS-CoV-2-infected individuals.

The adaptive immune response against SARS-CoV-2 is dominated by an antiviral T cell response and the production of neutralizing antibodies (Azkur et al. 2020; Moss 2022): The CD8^+^ T cell response is crucial for effective clearance of SARS-CoV-2 (Bergamaschi et al. 2021), and the (type 1) CD4^+^ T cell response is important for effective viral control (Notarbartolo et al. 2021), while antibodies play a critical role in virus neutralization but wane over time (Wu et al. 2007; Tang et al. 2011). Given their central role in the generation of immune responses and high population polymorphism, human leukocyte antigen (HLA) molecules are very likely candidates for explaining variation in COVID-19. Indeed, there is evidence that HLA genotype may affect COVID-19 disease outcome (Augusto and Hollenbach 2022). Variation in HLA molecules and viral antigens results in differential presentation of viral peptides on HLA molecules, thereby affecting the efficacy of the SARS-CoV-2 targeting immune response and COVID-19 outcome (Langton et al. 2021). Thus, some alleles were classified as risk and protective alleles. For instance, HLA-B*15:01 and HLA-DRB1*04:01 were associated with asymptomatic SARS-CoV-2 infection (Augusto et al. 2021; Langton et al. 2021). In contrast, HLA-A*11:01 and HLA-DRB1*09:01 were correlated with severe COVID-19 outcome (Wang et al. 2020; Khor et al. 2021; Anzurez et al. 2021).

The entire world is now free of very strict social restrictions because SARS-CoV-2 vaccines effectively induce neutralizing antibodies directed at the spike protein (Martínez-Flores et al. 2021), which is important for SARS-CoV-2 entry into host cells. However, vaccines put selection pressure on the virus, thereby promoting the evolution of immune escape variants (Cobey et al. 2021). Multiple major SARS-CoV-2 variants of concern (VOCs) emerged so far since the pandemic started: Alpha, Beta, Gamma, Delta, and Omicron (BA.1 to BA.5) (Harvey et al. 2021). The mutations in VOCs are often in the receptor-binding domain (RBD) of the spike protein, promoting faster infection of the target cells, and/or escape of antibody neutralization (Liu et al. 2021; Wang et al. 2021; Moss 2022). Especially the spike protein in the Omicron variant is highly mutated and escapes antibody immunity much more efficiently than the other VOCs (Cao et al. 2022). It is not yet known how the mutations affect T helper cell responses which are necessary for stable, long-lasting neutralizing antibody responses.

In this study, we aim to study spike (CD4^+^) T cell epitopes in silico and investigate the effect of vaccine selection pressure on epitope conservation and mutations in VOCs. Simulating a population with realistic HLA frequencies, we visualize the T helper cell immunity on the population level with respect to SARS-CoV-2 (and other human coronaviruses). With these results, we hope to gain insight into the potential need for a new (booster) vaccine to parallel SARS-CoV-2 immune evasion in the future.

## Results

### Most predicted T helper cell epitopes are experimentally verified in the IEDB

To investigate the difference between individuals in potential CD4^+^ T cell epitopes in the SARS-CoV-2 spike protein, we selected the highly polymorphic HLA-DRB1-restricted responses. Other HLA class II loci, e.g., HLA-DRA, are much less polymorphic and thereby would not contribute much to the diverse responses observed among people (Parham and Janeway 2015). We used the state-of-the-art NetMHCIIpan method (https://services.healthtech.dtu.dk/

service.php?NetMHCIIpan-4.1) (Reynisson et al. 2020; DTU Health Tech 2022) to predict CD4^+^ T cell epitopes. This method, based on an artificial neural network, is trained using two different datasets: eluted ligand (EL) and binding affinity (BA). The EL-trained model includes the likelihood of a peptide being produced in the cell and naturally presented by an HLA-DRB1 molecule, whereas the BA-trained model focuses on peptide-HLA binding affinity only. As expected, the EL-trained model predicted less coronavirus spike epitopes than the BA-trained model (Supplementary Fig. 1). To take peptide processing probability into account and not have an overestimation of possible CD4^+^ T cell epitopes, we focused our analysis on EL-based epitope predictions in the rest of the paper.

**Fig. 1:**
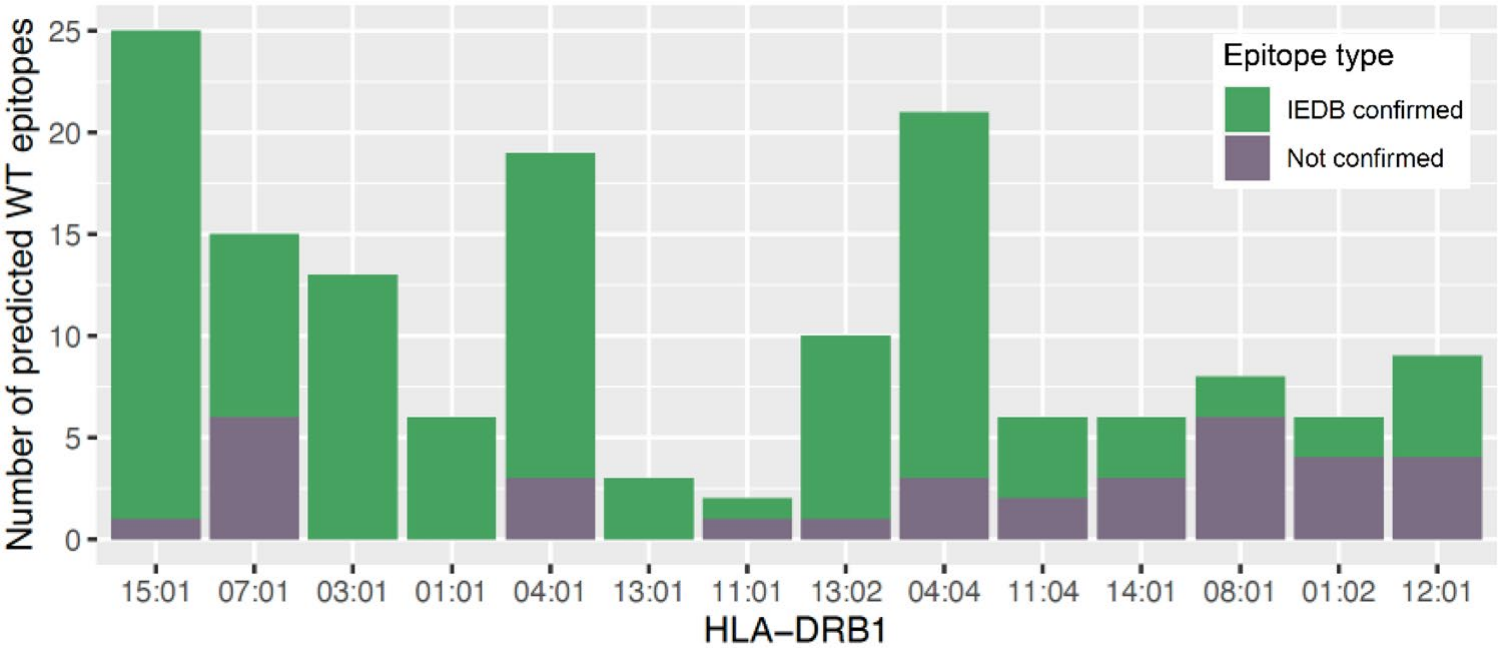
The majority of predicted T helper cell epitopes were confirmed in the IEDB. The number of predicted SARS-CoV-2 WT epitopes per HLA-DRB1 allele is plotted as bar plots. Predicted SARS-CoV-2 WT epitope cores were compared with experimentally confirmed DRB1-restricted T helper cell epitopes in the IEDB. The confirmed epitopes are shown in green and not confirmed ones are depicted in gray

To estimate the reliability of the in silico HLA-DRB1 epitope predictions, we investigated whether predicted SARS-CoV-2 WT epitopes were registered in the IEDB (www.iedb.org, Immune Epitope Database and Analysis Resource 2022) database as a T helper cell epitope. The percentage of predicted epitope cores that were part of an experimentally verified DRB1-restricted T helper cell epitope ranged between 25 and 100% per allele (Fig. 1). As expected, high frequency alleles generally showed a higher number of predicted epitopes than low frequency alleles because of higher data availability. In general, the number of experimentally verified epitopes seems to increase with allele frequency, though this correlation is not significant (Supplementary Fig. 2). We believe that this possible association reflects the performance of NetMHCIIpan being better for more common alleles than rare alleles, rather than being a novel, biologically relevant, finding. The top six most frequent alleles have a verified epitope percentage between 60% and 100%, indicating a relatively high prediction accuracy for these alleles. In conclusion, although SARS-CoV-2 is a novel virus, a far majority of predicted HLA-DRB1 restricted epitopes in the spike protein were experimentally verified, reflecting the intensity of research done on the coronavirus in the recent years (Reynisson et al. 2020; DTU Health Tech 2022).

### The number of predicted spike protein T helper cell epitopes varies per allele and little WT epitopes are lost in VOCs

**Fig. 2:**
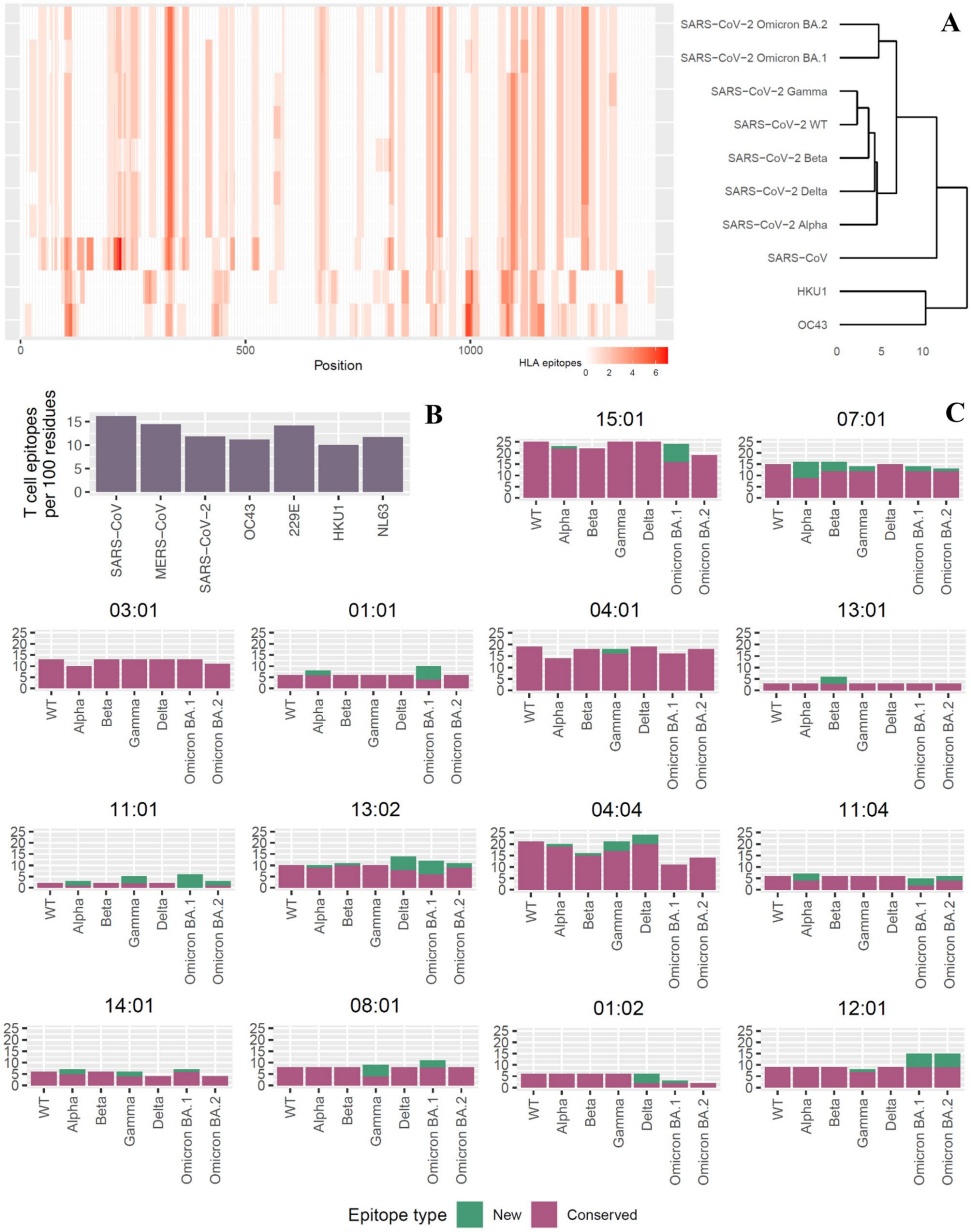
Predicted T helper cell epitopes in the spike protein of coronaviruses. **A** Number of predicted CD4 + T cell epitopes per HLADRB1 molecule in the spike protein (red scale). Positions on the *x*-axis indicate the position on the multiple made with the spike proteins from the viruses indicated in the figure (see also the “Methods” section). The number and position of epitopes in the coronaviruses were hierarchically clustered using complete linkage. **B** Number of predicted T helper cell epitopes per 100 amino acids per coronavirus. **C** Number of conserved (pink) and novel (green) T helper cell epitopes per allele per SARS-CoV-2 virus. HLA-DRB1 order in the figure is sorted using the allele frequencies

To visualize the distribution of T helper cell epitopes in the spike protein for the different coronaviruses, we predicted HLA-DRB1 restricted epitopes over the entire length of the spike proteins. The spike proteins of SARS-CoV, MERS-CoV, alpha HCoVs 229E and NL63, and beta HCoVs OC43 and HKU1 were included as they are orthologs to the to the SARS-CoV-2 spike protein. Predicted T helper cell epitopes were scattered across the spike protein, with a low epitope density in the middle of the protein and a high epitope density at the start and the end of the protein for SARS-CoV, SARS-CoV-2, and VOCs (Fig. 2A and Supplementary Fig. 3). In Fig. 2A, coronaviruses were hierarchically clustered based on the location of predicted CD4^+^ T cell epitopes in the spike protein. This clustering is in perfect agreement with the phylogeny of the coronaviruses (Fig. 2A) (Chan et al. 2015; Attwood et al. 2022). HKU1 and OC43 spike protein had a higher epitope density at the end of the protein, but not at the start of the protein. Most epitopes were predicted to bind one or two different HLA-DRB1 molecules, except for some outstanding universal epitopes such as one around position 315 for SARS-CoV, SARS-CoV-2, and VOCs and one around position 1000 for HKU1 and OC43. Interestingly, SARS-CoV spike had more predicted epitopes at the start of the protein than SARS-CoV-2 and VOCs, indicating that many mutations in this area of the protein may have resulted in loss of epitopes for SARS-CoV-2.

Next, we investigated the number of predicted T helper cell epitopes between SARS coronaviruses and HCoVs. As the length of the spike proteins varied between coronaviruses (1170–1355 amino acids), the total number of predicted spike epitopes was corrected for protein length. The number of T helper cell epitopes per 100 amino acids for 14 most common HLA-DRB1 molecules ranged between 10 and 15 for the coronaviruses (Fig. 2B). SARS-CoV spike contained the most predicted T cell epitopes, and HKU1 contained the least predicted T helper cell epitopes. Generally, there was little variety in the number of T helper cell epitopes between the coronaviruses.

To get more insight in the number of predicted T helper cell epitopes per HLA-DRB1 molecule and the appearance or disappearance of epitopes in the VOCs, we calculated the total number of epitopes of the SARS-CoV-2 WT for each allele and compared the amino acid sequences of all SARS-CoV-2 WT and VOC epitopes. Remarkably, HLA-DRB1 13:01 and 11:01 showed an exceptionally low number of predicted epitopes (2 to 6 epitopes, Fig. 2C). To determine whether this is due to a bias in our prediction method, we performed the same analysis on influenza A H1N1 and H3N2 hemagglutinin (Supplementary Fig. 4). When comparing the number of epitopes between the spike and hemagglutinin proteins, it is important to note that the spike protein is around twice as large as hemagglutinin and is therefore expected to have more CD4^+^ T cell epitopes. Without correcting for this, H3N2 and H1N1 hemagglutinin had 5 and 6 predicted epitopes for HLA-DRB1 13:01, which is higher than the number of epitopes for SARS-CoV-2 WT (Supplementary Fig. 4). Moreover, H3N2 and H1N1 hemagglutinin had 11 and 14 predicted epitopes for HLA-DRB1 11:01, which is also much higher than the number of epitopes for SARS-CoV-2 WT. These results suggest that the lack of potential epitopes for these two HLA-DRB1 molecules is not due to a prediction method bias, but it reflects the extent of differences between HLA-DRB1 molecules in response to SARS-CoV-2 spike protein.

Interestingly, some predicted epitopes that were originally present in SARS-CoV-2 WT were lost in VOCs, but this varied per HLA-DRB1 and per VOC. For example, up to 50% of original epitopes were lost in Omicron BA.1 for HLA-DRB1 13:02, 04:04, 11:04, and 01:02. New epitopes were gained as well, which mainly descended from mutated conserved epitopes (data not shown). Omicron BA.2 retained more conserved epitopes than BA.1, likely because of its different mutation profile. Overall, there was no excessive loss of original T helper cell epitopes in the VOCs, as reported by Tarke et al (2022).

#### Turnover of T helper cell epitope recognition at the population level

**Fig. 3:**
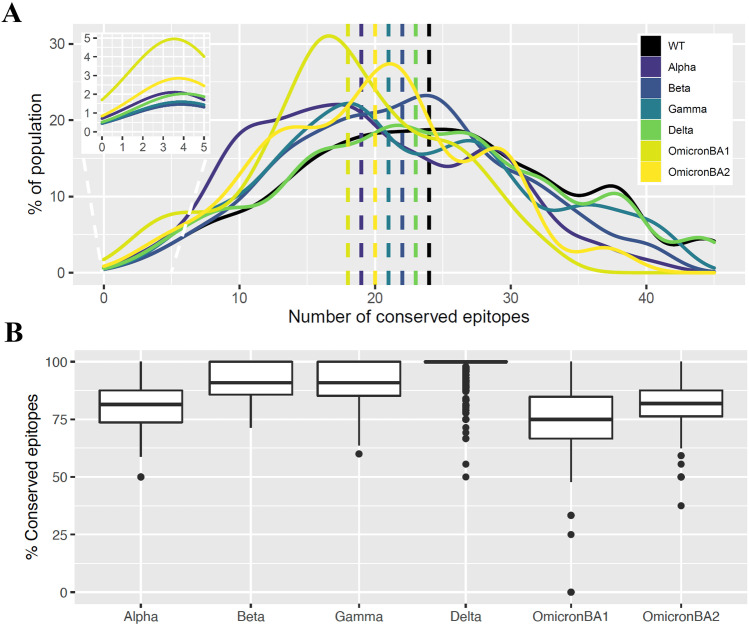
Predicted T helper cell epitope recognition in an artificial population. **A** Distribution of the number of conserved predicted epitopes per virus in an artificial population of 20 million individuals. **B** Percentage of original (WT) epitopes still recognized per VOC. All pairwise comparisons were statistically significant (*p* < 0.0001)

Because it is unclear whether subtle T helper cell epitope losses (Fig. 2B–C) have an impact on epitope recognition in a large population, we simulated a population of 20 million individuals with two assigned HLA-DRB1 alleles using realistic allele frequencies (downloaded from the National Marrow Donor Program, NMDP). The total number of predicted epitopes was then calculated per SARS-CoV-2 virus per individual. For all viruses, the number of epitopes recognized by an individual ranged between 0 and 45, with most of the population recognizing between 16 and 26 conserved epitopes depending on the virus (Fig. 3A). This number is in the range reported by Tarke et al. (2022), who tested four donors in depth for CD4 T helper cell responses after vaccination and found that each donor recognized 5–42 epitopes. Luckily only around 1% of the population had no predicted CD4 T helper cell epitopes in WT. The maximum number of recognizable epitopes in WT and Delta were 45 (recognized by 5% of the population), while the maximum number of recognizable epitopes in Omicron BA.1 was 35 epitopes, recognized by only 1% of the population. Strikingly, the percentage of the population recognizing zero epitopes was approximately doubled for Omicron BA.1 compared to the other viruses. Moreover, the simulated population recognized the least epitopes in Omicron BA.1 followed by Alpha and Omicron BA.2 (medians at 18, 19, and 20 epitopes, respectively). Taken together, these results suggest that the number of CD4^+^ T cell epitopes per individual seem to decrease with the recent VOCs, which might point to a possible escape from the immunity built up in the last 2.5 years. However, the median number of T helper cell epitopes remain high.

To put these data into the current day perspective, we analyzed the vaccination outcome in the simulated population with the WT SARS-CoV-2 spike protein, which the mRNA in the Comirnaty (BioNTech/Pfizer) vaccine is based on (European Medicines Agency 2021). The percentage of original epitopes still recognized in each VOC was then calculated. Half of the vaccinated population was predicted to recall less than 75% of CD4^+^ T cell epitopes when infected with Omicron BA.1 (Fig. 3B). In contrast, almost the entire population was predicted to generate full recall responses when infected with Delta. These results are in line with Fig. 2C, which shows that Omicron BA.1 lost the most HLA-DRB1 epitopes, while Delta generally kept almost all WT CD4^+^ T cell epitopes. Since the Omicron variant was first spotted in South Africa, we wanted to test whether this variant could have escaped even more CD4^+^ T cell responses in the local population. It is a challenge to obtain HLA frequencies for South African populations, as many studies made in this area have less than 160 individuals (http://www.allelefrequencies.net/). Therefore, we have used the African American HLA-DRB1 allele frequencies (Maiers et al. 2007; National Bone Marrow Program 2022) which are used as an approximation to simulate a South African population. The predicted CD4^+^ T cell epitopes in this population (Supplementary Fig. 5) are like ones shown in Fig. 3B, suggesting that the Omicron variant has not been specially adapted to the local population.

### Mutations occur mostly outside of CD4^+^ T cell epitopes

**Fig. 4:**
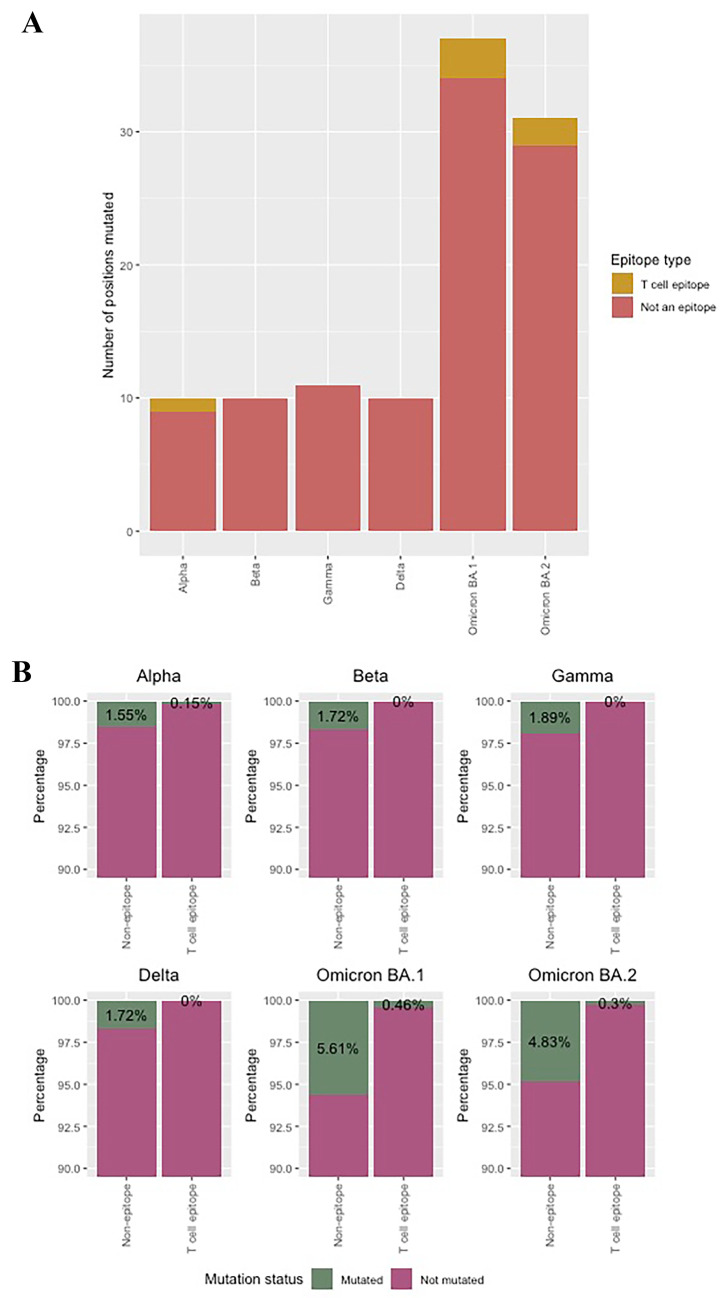
Mutation analysis of SARS-CoV-2 spike protein in SARS-CoV-2 VOCs. **A** Number of spike residues mutated in VOCs per epitope category: in non-epitope (gray) and in predicted T helper cell epitope (light pink). **B** Percentage of residues mutated (green) and not mutated (pink) within each epitope category. In all cases, the difference in the percentage of mutated positions were significantly different between T cell epitopes and non-epitopes (*p* < 0.001). The number of residues of the spike protein covered by T helper epitopes is 658, leaving a total of 651 positions for non-epitope category

To determine whether the occurrence of the mutations in T cell epitopes is different than the rest of the protein, we compared the number of mutations in different parts of the spike protein. To this end, we combined the predicted T helper cell epitopes of all 14 HLA-DRB1 alleles. Grouping the residues on the spike protein into two categories (non-epitope and T helper cell epitope), we found that most mutations occurred in spike regions that are predicted as non-epitopes (Fig. 4A). The number of mutations highly increased in Omicron BA.1 and BA.2 compared to the other VOCs, where Omicron BA.2 had less mutations than Omicron BA.1, which is in line with previous results showing higher epitope conservation in Omicron BA.2.

To test whether SARS-CoV-2 spike mutations occur more often in T helper cell epitopes, the percentage of mutated residues within the total number of spike residues of each category (non-epitope, or T helper cell epitope) was calculated. Interestingly, mutations occurred more often in non-epitopes than in T helper cell epitopes (*p* < 0.001 for all VOCs), indicating that predicted T helper cell epitopes are mainly present in conserved parts of the spike protein. In conclusion, SARS-CoV-2 spike mutations clearly occur less frequently in T helper cell epitopes. There might be two reasons explaining this observation. First, high polymorphism of HLA class II molecules in the population blocks the adaptation of the virus to escape T helper cell epitopes. Second, MHC molecules in general bind preferably hydrophobic peptides that are less likely to mutate (Ferrante & Gorski, 2007). Unfortunately, we cannot predict which factor plays the major role in conservation of the epitopes.

## Discussion

The aim of this study was to investigate the evolution of major T helper cell epitopes at the population level in SARS-CoV-2 VOCS. In summary, we demonstrated a general conservation of majority of T helper cell epitopes in all VOCs. However, it remains challenging to directly link predicted T helper cell epitope recognition to antibody immunity and vaccine efficacy. Our HLA-DRB1 epitope predictions showed that the number of predicted spike epitopes varies per allele (Fig. 2C). Especially HLA-DRB1 13:01 and 11:01 showed a dramatically low number of predicted epitopes. Therefore, we hypothesize that individuals with these DRB1 alleles may have a limited SARS-CoV-2 targeted CD4^+^ T cell response, which might result in a limited B cell response. However, Ragone et al. (2021) showed that the number of predicted HLA-DRB1-restricted epitopes was not correlated with short- and medium-term antibody titers. Still, Charonis et al. (2022) demonstrated a highly significant correlation between HLA-II binding affinity to spike and vaccine efficacy against VOCs. This study based their NetMHCIIpan (Reynisson et al. 2020; DTU Health Tech 2022) peptide binding predictions on 66 common HLA-II molecules in silico and accurately predicted a vaccine effectiveness of 71% against the Omicron BA.1 variant, which is close to the reported vaccine efficacy of 66% (Hayawi et al. 2021; Andrews et al. 2022). Interestingly, our simulated population results are in line with these predictions, as we demonstrated a median around 75% conserved epitopes recognized in a simulation of Omicron BA.1 infection (Fig. 3B). We believe that an important factor keeping T cell immunity rather robust against VOCs on a population scale is the polymorphism of HLA molecules (Parham and Janeway 2015; Moss 2022). Moreover, point mutations can be more detrimental for antibody responses than T cell responses, as it has been shown that wild-type-specific CD4^+^ and CD8^+^ T cells show cross-reactivity against the Omicron variant (Keeton et al. 2022).

It is difficult to accurately estimate the minimum number of predicted epitopes are required to generate a sufficient immune response. Although HLA-DRB1 epitope prediction accuracy was deemed high based on the IEDB confirmation percentage (Fig. 1), MHC class peptide binding predictions are clearly much more challenging than MHC class I predictions. Moreover, the number of predicted CD4 T cell epitopes is probably overestimated in this study, as there is a chance that a predicted epitope is false positive, not all presented peptides generate a T cell response, and optimally predicting the probability of peptide processing remains a challenge. Additionally, two individuals with the same HLA alleles could still harbor a different T cell repertoire and may therefore not recognize the same epitopes. Other factors impacting the T cell repertoire may be age, cross-reactivity acquired from previously encountered human coronaviruses, underlying (immune) diseases, or adverse effects from treatments like chemotherapy (Krishna et al. 2020). Consequently, it is likely that the percentage of individuals in our simulated population recognizing little or no T helper cell epitopes for Omicron BA.1 (up to 5%) is underestimated, suggesting that a significant proportion of the population might be lacking T helper responses following vaccination.

In this study, we used 14 most common HLA-DRB1 molecules in European populations (covering 90% of the population). Luckily many of the alleles we studied are also rather common in other ethnicities. For example, the alleles we used in this analysis makes up 57% in African and Hispanic populations and 44% in Asian populations (based on https://bioinformatics.bethematchclinical.org/, Maiers et al. 2007; National Bone Marrow Program 2022). Therefore, our overall results are likely to stay the same for other populations. Moreover, many alleles that are common in different populations are functionally similar. For example: DRB1*15:01 and 15:03 alleles are the most common alleles in Causation and African American populations. Out of 27 predicted strong binders for DRB1*15:01 in WT Spike protein, 24 are predicted also as strong binders for DRB1*15:03, and the remaining 3 are medium binders (within 1,5% of top binding peptides, results not shown). Given all these arguments, we believe that our analysis is a good overview of the effect of SARS-CoV-2 virus evolution on global CD4 T cell epitopes.

Unfortunately, it is still a big challenge to be able to predict B cell epitopes. When we used the best available tool to predict continuous epitopes, DiscoTope, (Immune Epitope Database and Analysis Resource 2022), http://tools.iedb.org/discotope/, which mainly predicts linear B cell epitopes but makes use of the protein structure to estimate surface accessibility of the predicted epitopes, we found that the IEDB contained a tenfold more experimentally verified B cell epitopes than we predicted (results not shown). Moreover, predicted continuous B cell epitopes covered a much smaller portion of the spike protein than the T helper cell epitopes. All these factors are, currently, limiting to study the evolution of B cell epitopes computationally. Still, we observed that even in continuous B cell epitopes, there are significantly more mutations than in CD4 T cell epitopes (results not shown), suggesting that there might be a strong selection pressure on SARS-CoV-2 to escape human B cell responses.

In conclusion, we demonstrated in silico that selection induced by vaccination worldwide has marginal effects on SARS-CoV-2 spike-specific CD4 T cell responses, while this might be not at all the case for B cell responses. Therefore, it might be worthwhile to consider inclusion of other less mutating SARS-CoV-2 proteins such as ORF3, NSP3, and the N protein in a future vaccine. Moreover, we have identified a few HLA-DRB1 molecules with surprisingly few potential epitopes in spike protein. Especially for those individuals carrying exclusively, these alleles including extra SARS-CoV-2 proteins in a vaccine would be very beneficial.

## Methods

### Viral protein sequences

Wild-type (WT) SARS, Middle East respiratory syndrome (MERS), and 229E and NL63 coronavirus spike protein sequences were retrieved from UniProt (UniProt Consortium 2022) using accession numbers UPI000018FE19 (SARS-CoV), NC_045512 (SARS-CoV-2/Wuhan-Hu-1), K9N5Q8 (MERS-CoV), P15423 (229E), and Q6Q1S2 (NL63). HCoV and VOC spike protein sequences were retrieved from NCBI Virus (National Center for Biotechnology Information 2022) using accession numbers YP_009555241 (OC43), YP_173238 (HKU1), UFQ05186 (SARS-CoV-2 Alpha variant/B.1.1.7), UJZ29611 (SARS-CoV-2 Beta variant/B.1.351), QRX39401 (SARS-CoV-2 Gamma variant/P.1), UJZ23640 (SARS-CoV-2 Delta variant/B.1.617.2), UIZ71506 (SARS-CoV-2 Omicron variant/BA.1), and UPH86598 (SARS-CoV-2 Omicron variant/BA.2). Influenza A virus hemagglutinin protein sequences were likewise retrieved from NCBI Virus using accession numbers YP_009118626 (H1N1/California 2009) and YP_308839 (H3N2/New York 2004).

## Multiple sequence alignment

Multiple sequence alignment was performed in RStudio (RStudio 2022) using the package “msa” (Bioconductor 2022) (version 1.27.2), running ClustalW with default parameters. The alignment was performed using the spike protein sequences of SARS-CoV, SARS-CoV-2, VOCs, OC43, and HKU1. Multiple sequence alignment resulted in a consensus sequence, of which the residue position numbers were used for later analysis of T helper and B cell epitope predictions.

## T helper cell epitope predictions

We have performed our analysis on the most common HLA-DRB1 alleles. HLA-DRB1 allele frequencies were obtained from the National Marrow Donor Program (NMDP) (Maiers et al. 2007; National Bone Marrow Program 2022). The 14 most prevalent HLA-DRB1 alleles in European Americans were 15:01 (14.4%), 07:01 (13.7%), 03:01 (12.9%), 01:01 (9.1%), 04:01 (9.1%), 13:01 (6.3%), 11:01 (5.6%), 13:02 (4.0%), 04:04 (3.6%), 11:04 (3.2%), 14:01 g (2.4%), 08:01 (2.4%), 01:02 (1.7%), and 12:01 g (1.5%). The likelihood of peptides to be naturally presented by HLA-DRB1 alleles was then predicted with NetMHCIIpan4.1 (Reynisson et al. 2020; DTU Health Tech 2022) using a model trained on an extensive eluted ligand mass spectrometry (EL) dataset. We used a fixed peptide length of 15 residues (15mers) to predict epitopes from coronavirus spike proteins and influenza A hemagglutinin proteins. There are three main reasons for using 15 as predicted peptide length. First, changing the peptide length has hardly any effect on our results: Peptides of length 12 are hardly ever predicted as binders. Moreover, very long peptides, e.g., 18mers, are also not predicted as likely ligands for these molecules. For the other peptide lengths, e.g., 13–17, the number of predicted strong binders per HLA-DRB1 molecule does not change. Often with 15mers, the maximum number of binders are obtained (results not shown). Second, NetMHCIIpan method that we used is trained with peptides in the range 13–21; however, most of the training data is of length 15. It is most likely that the performance of the method is the most optimal for 15mers. The web server of this method, therefore, by default, digests input proteins into 15mer peptides. Finally, we have also analyzed the length of over 800 known MHC class II SARS-CoV-2 epitopes from IEDB database and found that many of the epitopes are 14, 15, 16, or 17mers. Therefore, peptide length of 15 is a good estimate for an average CD4 T cell epitope length.

All peptides with a rank score of top 1% or lower were identified as strong binders or potential T helper cell epitopes. The total number of T helper epitopes in SARS coronaviruses and HCoVs was corrected for protein length. The number of conserved and new epitopes in VOCs was determined by comparing the sequences of WT and VOC epitopes with the same consensus start position. A VOC peptide was classified as “conserved” when it had the exact same amino acid sequence as the WT peptide or “new” when it had a rank score of 1% or lower but did not have the same sequence or position as the WT peptide. Data scaling was performed using min–max normalization. Hierarchical clustering of coronaviruses was performed following the “complete” method and based on the location of predicted CD4^+^ T cell epitopes in the spike protein.

## Confirmation of predicted T helper cell epitopes in the IEDB

Predicted epitopes were confirmed using the data from the Immune Epitope Database (IEDB, www.iedb.org) (Immune Epitope Database and Analysis Resource 2022. The IEDB contained 113 experimentally verified DRB1-restricted T helper cell epitopes on the 2nd of June 2022. These epitopes were verified with a variety of methods: MHC binding assays, multimer/tetramer binding assays, T cell-APC binding assays, and/or biological activity assays measuring cytokine release, activation, or degranulation. A predicted epitope was counted as “confirmed in the IEDB” if the predicted epitope core was part of an experimentally verified epitope in the IEDB.

## Simulated population

A population of 20 million individuals was simulated by randomly assigning two HLA-DRB1 alleles to each individual conforming to European American HLA-DRB1 allele frequencies. The total number of SARS-CoV-2 T helper cell epitopes was then calculated per individual. If a peptide was presented by two HLA class II molecules of an individual, it was counted once. The percentage of conserved SARS-CoV-2 epitopes recognized in a simulated “vaccinated” population was calculated by dividing the number of conserved VOC epitopes by the number of SARS-CoV-2 epitopes in the WT per individual.

## Mutation analysis

VOC mutations were obtained from the GISAID Mutation Tracker (Outbreak.info 2022). Based on its position in the spike protein, a mutation was classified as not present in an epitope or present in a T cell epitope. The number of positions mutated per VOC in each category was calculated. For statistical analysis, the percentage of the number of mutations within a total number of spike residues that are either a non-epitope (651 residues) or a T helper cell epitope (658 residues) was calculated.

## Visualization and statistical analysis

Graphs were created in R using the package “ggplot2” (Wickham 2016). Statistical analysis on the simulated population was performed using the Mann–Whitney *U* test. Statistics on the mutation analysis were performed with the Fisher’s exact test. Asterisks in the figures of this report are indicative of statistical significance: *p < 0.05, **p < 0.01, ***p < 0.001, and ****p < 0.0001.

## Supplementary Information

Below is the link to the electronic supplementary material.

Supplementary file1 (DOCX 858 KB)

## Data Availability

The scripts and data used to generate the figures of this paper will be available upon request. Please send an email to c.kesmir@uu.nl
