# Supplementary material for: Evolution of SARS-CoV-2-specific CD4+ T cell epitopes
Source: Immunogenetics. 2023 Jan 31;75(3):283–93. doi: 10.1007/s00251-023-01295-8 (PMC9887569; doi:10.1007/s00251-023-01295-8)
Supplement: Supplementary file 1 — Supplementary file1 (DOCX 858 KB) [file 251_2023_1295_MOESM1_ESM.docx]

**S1. Eluted Ligand versus Binding Affinity-based HLA-DRB1 epitope prediction**


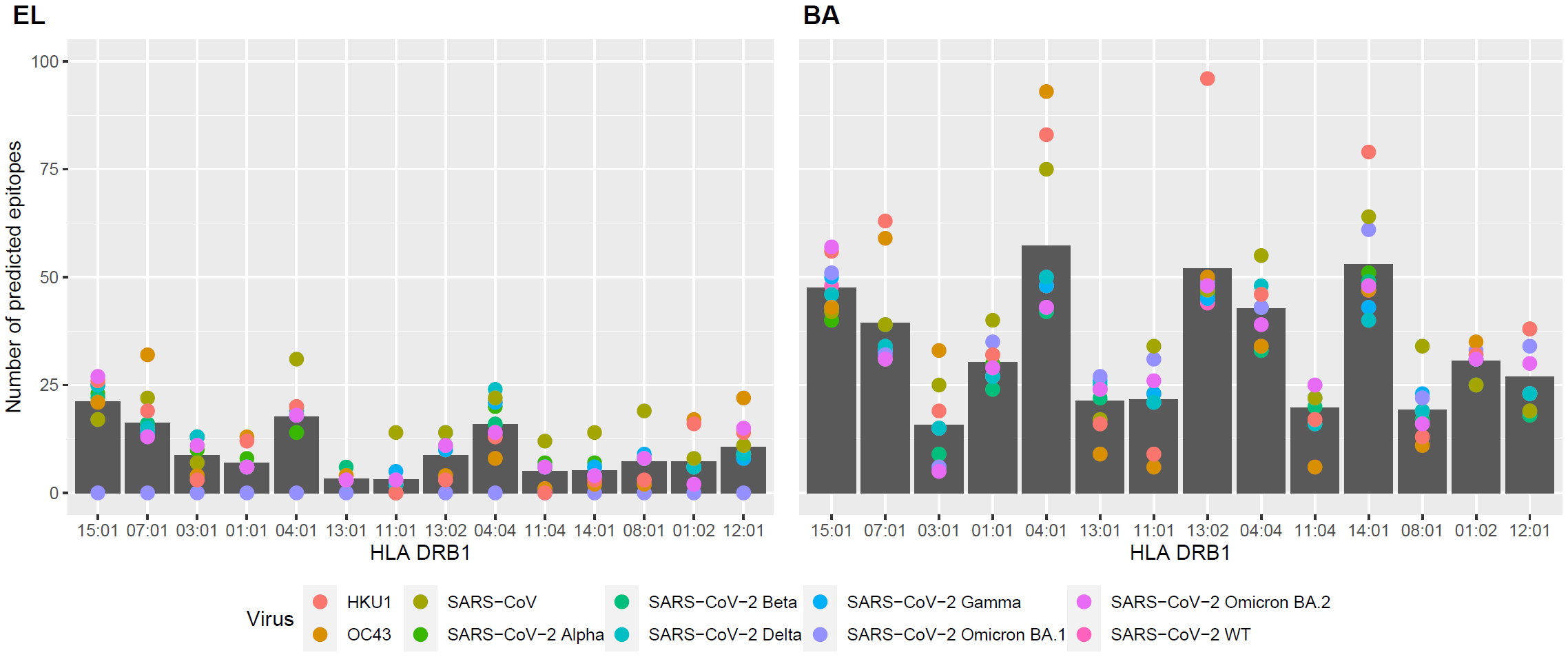


S1. Eluted Ligand versus Binding Affinity-based HLA-DRB1 epitope predictions**.** The number of predicted epitopes is shown per HLA-DRB1. HLA-DRB1 molecules are sorted based on their allele frequencies in the population.

**S2. Number of predicted epitopes in the SARS-CoV-2 spike protein plotted against HLA frequency.**

S2. Number of predicted epitopes in the SARS-CoV-2 spike protein plotted against HLA frequency**.** Trend line (blue) with 95% confidence interval. P-value was calculated with spearman correlation test in R.


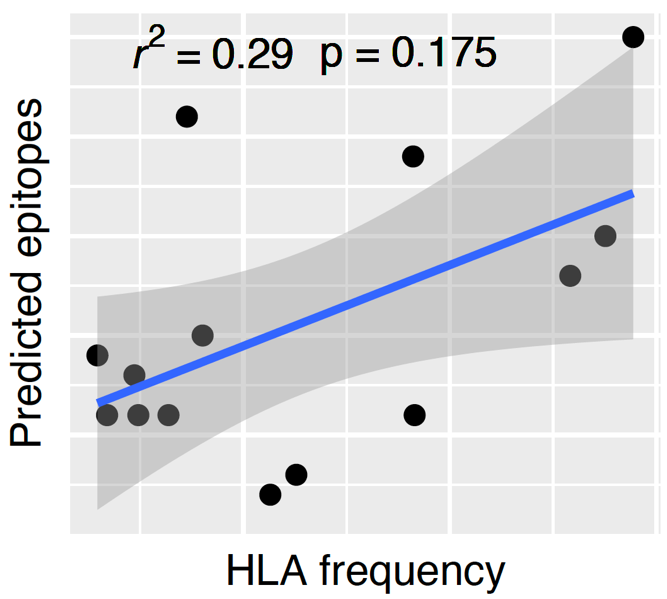

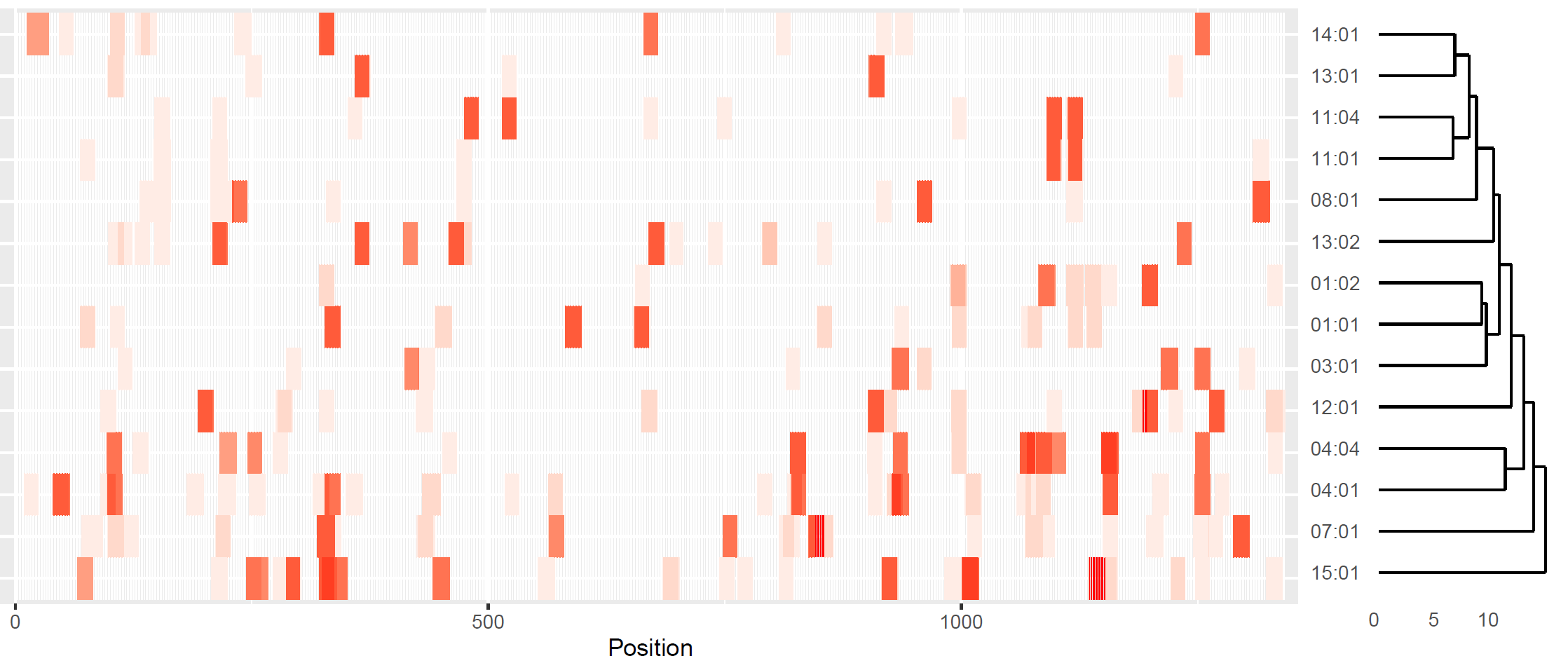

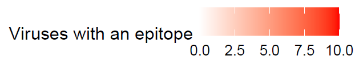


S3. Number of coronavirus epitopes per HLA-DRB1 molecule for each position in the spike protein**.** Number of coronaviruses (SARS-CoV, SARS-CoV-2 WT, Alpha, Beta, Gamma, Delta, Omicron BA.1, Omicron BA.2, HKU1, and OC43) with a predicted epitope per HLA-DRB1 molecule in the spike protein (red scale). Positions on the x-axis indicate the position on the multiple sequence alignment made with the spike proteins from the viruses. The number and position of epitopes for the HLA-DRB1 molecules were hierarchically clustered using complete linkage.
